# Presplicing factors associate with UpA-rich RNA and limit flavivirus replication

**DOI:** 10.1016/j.mocell.2026.100404

**Published:** 2026-10-05

**Authors:** Joyce W.M. van Bree, Kristel Doets, Carmen van de Waterbeemd, Sjef Boeren, Monique M. van Oers, Gorben P. Pijlman, Jelke J. Fros

**Affiliations:** 1 Laboratory of Virology, Wageningen University & Research, P.O. Box 16, Wageningen, 6700 AA The Netherlands; 2 Laboratory of Biochemistry, Wageningen University & Research, Postal address: Box 8128, Wageningen, 6700 ET, The Netherlands

**Keywords:** RNA affinity purification-mass spectrometry, RNA-binding proteins, Spliceosome, Splicing machinery, UpA dinucleotide suppression

## Abstract

The RNA of all living organisms and most viruses exhibits pervasive suppression of uracil-adenine (UpA) dinucleotides, yet the underlying molecular mechanisms remain poorly understood. Here, we use RNA affinity purification coupled with mass spectrometry to identify diverse host factors that preferentially bind UpA-high RNA sequences. The identified proteins include the canonical antiviral proteins IFIT1 and OAS1, as well as multiple components of the U1 and U2 small nuclear ribonucleoproteins (snRNPs), core elements of the splicing machinery. These splicing factors display increased affinity for artificially UpA-enriched RNA and the West Nile virus (WNV) 3’ UTR, which naturally contains higher frequencies of UpA dinucleotides. The functional relevance of splicing factor interactions was demonstrated by pharmacological inhibition of early splicing events, which partially restored the replicative fitness of synonymously recoded and otherwise attenuated UpA-high mutants of Zika virus and WNV. Together, these findings reveal a novel antiviral role for splicing factors, linking their preferential association with UpA-rich RNA to the restriction of UpA-high viruses. Given the evolutionary conservation of the splicing machinery, our results suggest that splicing factor-mediated recognition may contribute to maintain the universal suppression of UpA dinucleotides in host and viral RNA.

## Graphical Abstract

**Figure:**
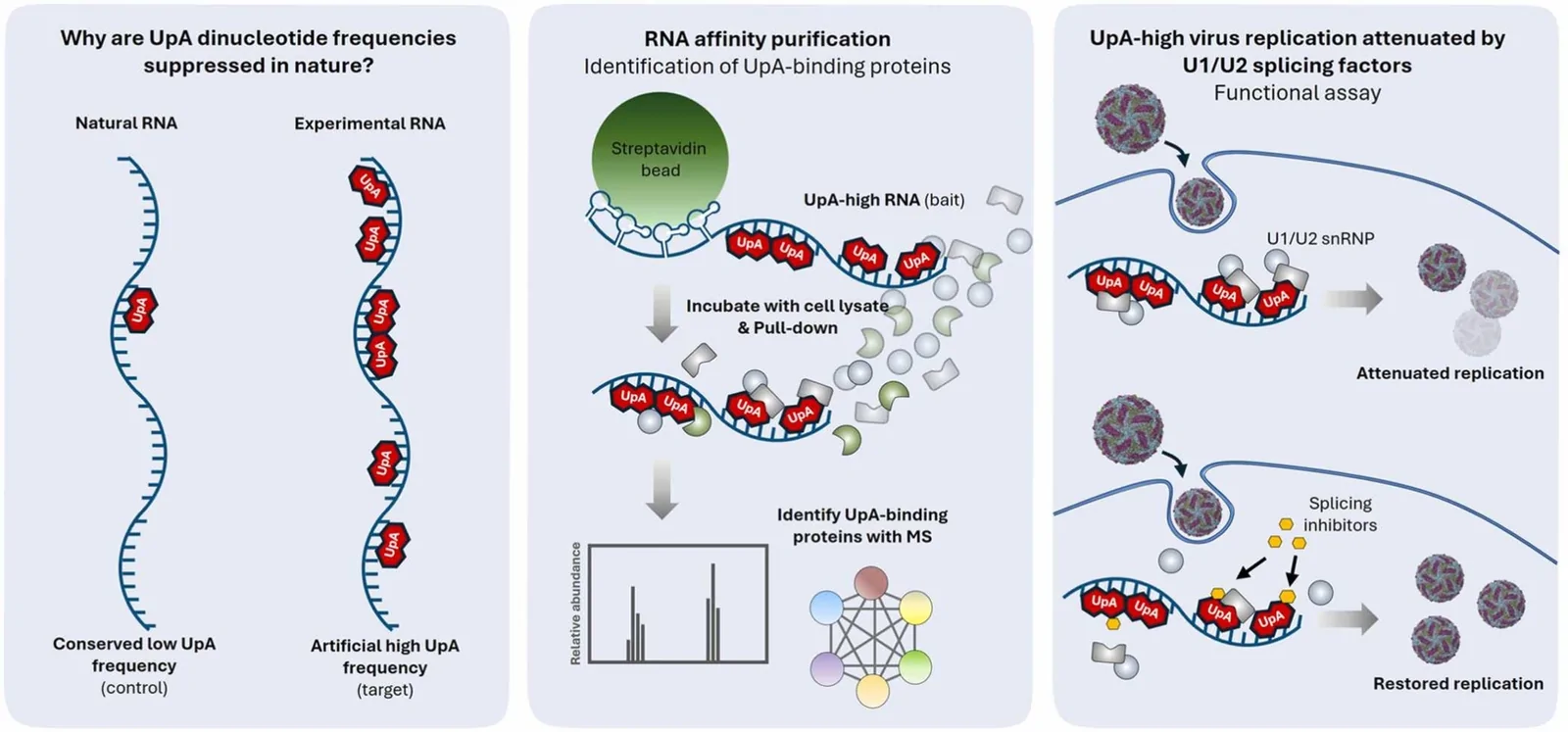

## BACKGROUND

RNA molecules sit at the heart of cellular life. Beyond their classical role as messenger between DNA and protein, RNAs function as regulatory molecules that modulate transcription, translation, and RNA decay pathways (reviewed in (Cao et al., 2024)). Interactions with RNA-binding proteins through conserved sequence features are fundamental to RNA fate. Consequently, the selective pressure to either retain or avoid specific sequence features is likely to have shaped nucleotide composition throughout evolution.

One of the most striking biases in RNA nucleotide composition is the universal under-representation of UpA dinucleotides, observed across prokaryotic and eukaryotic genomes (Beutler et al., 1989, Burge et al., 1992, Kariin and Burge, 1995, Nussinov, 1984). This suppression of UpA dinucleotides extends beyond cellular organisms to many viral species (Simmonds et al., 2013, Rima and McFerran, 1997). Experimental evidence underscores the functional importance of this suppression, as artificially increasing the UpA frequency in viral RNA significantly impairs replication of diverse viruses, including echovirus 7 (Atkinson et al., 2014; Fros et al., 2017; Tulloch et al., 2014), poliovirus (Burns et al., 2009), Zika virus (ZIKV) (Fros et al., 2021), and West Nile virus (WNV) (van Bree et al., 2025). While previous studies have implicated the zinc-finger antiviral protein (ZAP) in UpA-dependent attenuation of ZIKV (Fros et al., 2021, Odon et al., 2019), this phenotype has not been observed for WNV (van Bree et al., 2025), and structural and transcriptome-wide binding studies indicate that ZAP preferentially recognizes CpG-rich rather than UpA-rich RNA (Meagher et al., 2019, Takata et al., 2017), leaving the molecular basis of UpA recognition unresolved.

Despite the universal suppression of UpA dinucleotides across all types of cellular RNA and that of cytoplasmic RNA viruses, the mechanisms driving this selection remain incompletely understood. Several hypotheses have been proposed, including the stop codon hypothesis, which suggests that UpA suppression may reduce the likelihood of premature translation termination upon mutation (Duan and Antezana, 2003; Karlin and Burge, 1995; Trotta, 2016). However, this hypothesis cannot fully explain universal UpA suppression, as UpA suppression also occurs in noncoding regions and in organisms with different codon preferences (Simmonds et al., 2013). Another hypothesis is that the relatively low thermodynamic stability of UpA dinucleotides in RNA contributes to selection against this dinucleotide (Ghosh et al., 2023). Other proposed mechanisms involve the recruitment of AU-rich element (ARE)-binding proteins that promote mRNA degradation (Al-Saif and Khabar, 2012; Brewer et al., 2004; Duan and Antezana, 2003), or RNase L-mediated cleavage after UpA and UpU dinucleotides (Anderson et al., 2011; Kodym et al., 2009). Yet these mechanisms do not specifically target UpA dinucleotides or are restricted to vertebrates (Chu et al., 2023), failing to explain the selective and universal suppression of UpA dinucleotides across all domains of life.

Our study aims to elucidate the molecular mechanisms underlying the universal suppression of UpA dinucleotides by identifying human proteins that interact with synthetically UpA-enriched and naturally occurring viral UpA-high RNA sequences. Through comparative RNA affinity purification assays, we demonstrate that UpA-rich sequences selectively recruit host proteins involved in RNA metabolism, particularly components of the cellular splicing machinery. This connection between splicing factors and UpA-rich RNA provides a potential mechanism underlying the evolutionary pressure that has maintained UpA suppression from bacteria to humans and across viral genomes.

## MATERIALS AND METHODS

### Cells and Viruses

African green monkey kidney Vero E6 (ATCC CRL-1586), Vero (ATCC CCL-81), and human A549 cells (ATCC CCL-185) were maintained in completed Dulbecco’s modified Eagle medium (DMEM; Gibco) supplemented with 10% (v/v) heat-inactivated fetal bovine serum (FBS; Gibco), 100 U/ml penicillin, and 100 µg/ml streptomycin (pen/strep; Sigma-Aldrich). Cells were cultured at 37ºC in a humidified atmosphere containing 5% CO_2_. All cell lines were passaged biweekly.

The viruses used in this study were generated from pCC1-BAC plasmids containing an SP6 promoter-driven ZIKV BeH819015 isolate from Brazil (GenBank accession no. KU365778.1) and a pCC1-BAC containing a CMV promoter-driven WNV isolate 578/10 from Hungary (GenBank accession no. KC496015.1), as described previously (Mutso et al., 2017, Szentpáli-Gavallér et al., 2016). The production of infectious virus and construction of recoded ZIKV and WNV mutants was described previously (Bree et al., 2025; Fros et al., 2021).

### Construction of RNA-Aptamer Plasmids

Aptamer constructs were designed based on a previously described strategy in which bait RNAs are fused to 4 S1m aptamers, enabling affinity capture on streptavidin-coated beads for isolation of associated RNA-binding proteins (Göertz et al., 2019; Leppek and Stoecklin, 2014). Bait RNA sequences were cloned downstream of 4 S1m aptamers using various unique restriction sites present in pJET1.2-T7–4xS1m plasmid. Bait RNA sequences were based on a 1,653-nucleotide region of the ZIKV genome. The design of these ZIKV-derived bait sequences has been described in detail previously, and sequences are available (Supplementary File 1) (Fros et al., 2021). In short, 3 variants were generated: (1) a wild-type sequence with naturally low UpA dinucleotide content (UpA-low), (2) a sequence with artificially elevated UpA frequency (UpA-high), and (3) an artificial control sequence with UpA content similar to wild-type (UpA-low_a). All bait sequences were amplified using Q5 high-fidelity DNA polymerase (NEB) with forward (FW) and reverse (RV) primers containing *Xma*I and *Nsi*I overhangs (FW: 5’-CCCGGGCGTCTCCAGCGCCATACTGTCGCGGACCGC-3’ and RV: 5’-ATGCATCGTTCAAGAATCCAAGGGCTTCGAACTC-3’). Both the amplified bait sequences and pJET1.2-T7–4xS1m plasmid were digested with *Xma*I and *Nsi*I (New England Biolabs), gel-purified (GE Healthcare Illustra GFX PCR DNA and Gel Band Purification Kit), and ligated with T4 ligase (Promega). The pJET1.2-T7–4xS1m plasmids with the WNV nonstructural protein 2A (NS2A) and 3’ untranslated region (3’ UTR) sequences were generated using a similar cloning approach, but with different restriction enzyme combinations. The NS2A region was amplified from pBeloBAC-WNV using primers (FW: 5′-GGCATATGTACAACGCCGACATGATTGATCCTTTTCA-3′ and RV: 5′-GGGAATTCTAATCTCTAGACTGTACACATCAAGGTTTAGGCATTTTAGTCCA-3′), followed by digestion with *Nde*I and *Xba*I (New England Biolabs). The 3′ UTR region was amplified using primers (FW: 5′-CCCCCCGGGGACACTGTTTTGTAAATAATAAAGCTGTATTGAGTAGT-3′ and RV: 5′-CCCTCTAGAGATCCTGTGTTCTAGCACCAC-3′) and subsequently digested with *Xba*I and *Xma*I (New England Biolabs). The digested fragments were ligated into the appropriately digested pJET1.2-T7–4xS1m vector.

### RNA Synthesis

The 4xS1m aptamer and downstream bait sequences were PCR-amplified using GoTaq DNA polymerase (Promega) and FW (5’-TAATACGACTCACTATAGGGAGAGGTACCGG-3’) and RV (5’-AGATCCTGTGTTCTAGCACCACCAG-3’) primers. PCR fragments were purified by phenol-chloroform extraction, and 1 µg of purified DNA was used as template for *in vitro* RNA transcription using T7 RNA polymerase (New England Biolabs), according to the manufacturer’s protocol. Following T7 transcription, RNA samples were treated with DNase (Promega) and used as input for RNA affinity purification, according to the manufacturer’s protocol.

### Lysate Preparation

For RNA affinity purification, A549 cells were selected because they mount a robust innate immune response, allowing infection-induced changes in the RNA-binding proteome to be investigated. Cells were either mock-treated or infected with ZIKV at a multiplicity of infection (MOI) of 5 and incubated for 24 hours prior to harvesting. Per RNA affinity purification sample, ∼25 x 10^6^ A549 cells were harvested. Culture medium was aspirated, and cells were washed once with phosphate-buffered saline (PBS, Gibco). Cells were detached by scraping using cell scrapers (Greiner Bio-One) and pelleted by 500 RCF. Cells were washed twice with PBS, and pellets were stored at −80°C up to 1 week. Cells were lysed in 150 μl of homemade 2 x lysis buffer (2 x SA-RNP lysis buffer; 40 mM Tris-HCl [pH 7.5], 300 mM NaCl, 3 mM MgCl_2_, 20 µM ZnCl_2_, 4 mM DTT, 4 mM vanadyl ribonucleoside complex RNase inhibitor [VRC; New England Biolabs], and 2x Complete protease inhibitor cocktail [Roche]) supplemented with 2% (v/v) nonidet P40 (NP-40) by incubation on ice for 20 minutes. Cell debris was removed by centrifugation at 20,800 RCF for 15 minutes.

### RNA Affinity Purification

Per pull-down reaction, 100 μl of 50% Streptavidin Sepharose High Performance bead slurry (SA beads; GE Healthcare) was aliquoted in Low Protein Binding tubes (ThermoFisher Scientific). Beads were washed 3 times with 1 x SA-RNP lysis buffer (2 x SA-RNP lysis buffer diluted 1:1 with dH_2_O). After the final wash, 50 μl of 1 x SA-RNP lysis buffer was added to the beads to create a 50% slurry in 2 x SA-RNP lysis buffer. A total of 50 μl of DNase- treated *in vitro-*transcribed RNA was dissolved up to a volume of 100 μl in 2x SA-RNP lysis buffer. RNA was renatured by incubation at 56°C for 5 minutes, followed by 37°C minutes for 10 minutes and finally at room temperature for 2 minutes. Renatured RNA was added and coupled to the SA beads by incubation for 2.5 hours at 4°C with overhead rotation. Beads displaying the RNAs were collected by centrifugation at 100 RCF for 2 minutes, the unbound RNA mixture was removed, and 125 μl of lysate was supplemented with 80 U of RNasin (Promega) and added to the beads. To allow for RNA-protein complexes to be formed, beads with added lysates were incubated for 1 hour at 4°C under overhead rotation. After incubation, beads were collected by centrifugation at 100 RCF for 1 minute at 4°C and washed 4 times with 1 ml of homemade SA-RNP wash buffer (20 mM Tris-HCl [pH 7.5], 300 mM NaCl, 5 mM MgCl_2_, 10 µM ZnCl_2_, 2 mM DTT, 2 mM VRC, and 1 x Complete protease inhibitor cocktail [Roche]). A final wash was performed with 1 ml of 50 mM ammonium bicarbonate (ABC; Sigma-Aldrich). Finally, 90 μl of 50 mM ABC was added to the samples.

### Peptide Preparation and Identification via Mass Spectrometry

RNA pull-down samples (90 μl) were reduced by the addition of 10 μl of 150 mM DTT and incubation at 45°C for 30 minutes under gentle shaking. Proteins were alkylated by addition of 10 μl of 200 mM iodoacetamide (ThermoFisher Scientific) and incubation at room temperature for 30 minutes under gentle shaking. Subsequently, 10 μl of 200 mM cysteine (Sigma-Aldrich) was added, followed by 10 μl of sequence-grade trypsin (Roche, 1:10 diluted in ABC). Samples were digested overnight at room temperature under gentle shaking. The digestion was stopped by acidification with 10% trifluoro acetic acid to pH < 3. Peptides were concentrated and desalted using homemade µColumns as described previously (Rappsilber et al., 2007). The method was modified by adding a 4 μl of a 50% slurry LichroPrep C18 beads (Merck 1.09303, 50% v/v in methanol) on top of the C18 Stage tips (Affinisep SPE-Disks-Bio-C18-100.47.20).

Peptides were analyzed by nano LC-MS/MS coupled to an Orbitrap Exploris 480 mass spectrometer. Peptides were separated using a 50-minute acetonitrile gradient from 9% to 34% in 0.1% formic acid. MS and MSMS AGC targets were set to 300%, 100%, respectively, or maximum ion injection times of 50 ms (MS) and 30 ms (MSMS) were used. HCD-fragmented (Isolation width 1.2 *m/z*, 28% normalized collision energy) MSMS scans in a cycle time of 1.1 seconds of the 25 most abundant 2 to 5+ charged peaks in the MS scan were recorded in data-dependent mode (Resolution 15,000, threshold 5e4, 15 seconds of exclusion duration for the selected *m/z* +/- 10 ppm). Raw data were processed using MaxQuant software package (version 2.0.3.0) using the Human proteome (UP000005640_20_400_2019) and ZIKV BeH819015 isolate polyprotein sequences as reference databases. The mass spectrometry proteomics data have been deposited to the ProteomeXchange Consortium via the PRIDE partner repository with the dataset identifier PXD066653 (Perez-Riverol et al., 2025).

### Perseus, Panther, and STRING Analysis

The ProteinGroups MaxQuant result was filtered and analyzed using Perseus (version 1.6.15.0). Label-free quantification (LFQ) intensities were filtered to include only proteins identified by at least 2 peptides, with at least one unique and one unmodified peptide, and a false discovery rate (FDR) < 1% at both the protein and peptide level. Reversed hits were removed and LFQ values were log10-transformed. Data were grouped per condition and strictly filtered to retain proteins with at least 2 valid LFQ values in at least 1 group.

For differential binding analyses, proteins significantly enriched between RNA baits were identified using Student's *t*-tests (*P* < 0.05). However, because only a limited number of proteins reached statistical significance when directly comparing the artificial UpA-high and UpA-low RNA baits, this dataset was primarily used for exploratory pathway analyses. For these analyses, proteins showing at least a 2fold higher LFQ intensity for the UpA-high bait compared with both UpA-low control baits were included for Gene Ontology (GO) and STRING network analyses. This threshold was selected to identify broader biological patterns that were not apparent from significance testing alone due to the limited overlap between experimental conditions (Supplementary File 2).

### Cytotoxicity Testing

The compounds SRPK inhibitor (SRPIN340; Sigma-Aldrich), isoginkgetin (ISOGK; Sigma-Aldrich), and splitomycin (Sigma-Aldrich) were dissolved in dimethyl sulfoxide (DMSO) at 100 mM and stored at −20°C. Vero cells were seeded in 96-well plates at a confluency of ∼70%. Compounds were added to the medium at the indicated concentrations, and the cells were incubated for 3 days. After 3 days, the medium was replaced with MTT solution (5 mg/ml in PBS, filtered; ThermoFisher Scientific) diluted 1:1 in serum-free medium, and the cells were incubated for 4 hours. After incubation, the MTT solution was replaced with 100 μl of DMSO. Cells were placed back in their respective incubator for an additional 15 minutes. Absorbance was measured at 590 nm using a microplate reader (Tecan infinite 200).

### Splicing Inhibition Validation

Vero cells were incubated with 20 µM SRPIN340, 20 µM ISOGK, or 250 µM splitomycin for 72 hours. Total RNA was isolated using TRIzol reagent (Invitrogen) according to the manufacturer's protocol. RNA concentration and purity were determined using Qubit RNA BR Assay Kit (ThermoFisher Scientific). Equal amounts of RNA from each sample were reverse-transcribed into cDNA using SuperScript III Reverse Transcriptase (Invitrogen) with random hexamer primers (Roche) following the manufacturer's instructions. Quantitative PCR (qPCR) was performed to assess splicing efficiency using exon-exon junction-spanning primers targeting GAPDH (FW: 5′-AGAAGGCTGGGGCTCATTTG-3′, RV: 5′-AGGGGCCATCCACAGTCTT-3′) and PowerTrack SYBR Green MasterMix (ThermoFisher Scientific) on a CFX96 Real-Time PCR instrument (Bio-Rad).

### Splicing Inhibition and Virus Infections

Vero cells were seeded in a 96-well to achieve ∼70% confluency. The compounds SRPIN340, ISOGK, and splitomycin were added to the medium at indicated concentrations 2 hours prior to infection with ZIKV or WNV variants (MOI = 0.1). After 2 hours of incubation, the cells were washed with PBS, and fresh medium containing the respective compounds was added. Infected cells were incubated for 2 days, after which supernatant samples were collected from each well and analyzed for the presence of infectious virus via end-point dilution assays (EPDA).

### siRNA-Mediated Knockdown and Virus Infection

A549 cells were seeded in a 24-well plate to achieve ∼50% confluency. Cells were transfected twice, 48 hours apart, with either siRNAs targeting SNRNP70 (si70K; Dharmacon, Horizon discovery) or a nontargeting control siRNA (siNT; Dharmacon, Horizon discovery) using DharmaFECT transfection reagent (Horizon discovery) according to the manufacturer’s instructions. A final siRNA concentration of 100 nM was used for each transfection. Three hours after the second transfection, cells were infected with WT or UpA-high ZIKV (MOI = 0.1). After 2 hours of incubation, the inoculum was removed, cells were washed with PBS, and fresh medium was added. Infected cells were incubated for 2 days, after which supernatants were collected for quantification of infectious virus by EPDA. Knockdown efficiency was verified by Western blot analysis.

### Western Blot Analysis

To verify SNRNP70 knockdown, cell lysates were prepared in 1× SDS loading buffer and heat-denatured at 95°C for 15 minutes. Proteins were separated by SDS-PAGE on Mini-PROTEAN TGX Any kD precast gels (Bio-Rad), transferred to nitrocellulose membranes using the Invitrogen Power Blotter system, and probed with antibodies against SNRNP70 (Abcam, ab83306; 1:900) and GAPDH (Sigma-Aldrich, G9545; 1:5,000). Bound antibodies were detected using an alkaline phosphatase-conjugated goat anti-rabbit secondary antibody (Invitrogen; 1:2,500) and NBT/BCIP substrate (Roche).

### Virus Quantification

The infectious virus titer was quantified using the median tissue culture infectious dose (TCID_50_), determined via EPDAs on Vero E6 cells. Confluent Vero E6 cell cultures were detached with Trypsin-EDTA (Gibco) and subsequently resuspended in complete DMEM to achieve a final concentration of 2.5 x 10^5^ cells/ml. Serial 10-fold dilutions of viral stocks and samples were prepared in DMEM supplemented with 10% FBS, 100 U/ml penicillin, and 100 µg/ml streptomycin. The cell suspension was mixed with the virus dilutions in a 1:1 ratio and plated in 6-fold into microtiter plates (Nunc, Sigma-Aldrich). Viral titers were determined by observing the presence of cytopathic effects under a microscope 3 to 4 days post infection, using the Reed-Muench method.

### Immunofluorescence Assay

Cells were first fixed with 4% paraformaldehyde (PFA; Acros, ThermoFisher Scientific) in PBS for 10 minutes at room temperature. Next, cells were permeabilized (0.1% SDS in PBS for 10 minutes at RT) and blocked with 0.5% skimmed milk powder (ELK, Campina) in PBS for 30 minutes. Primary antibodies against SNRNP70 (ab83306; Abcam) and dsRNA (clone 3G1.1; (Harrison et al., 2020)) were diluted 1:500 in 0.5% ELK/PBS and incubated with the cells for 1 hour at 37°C. Secondary antibodies goat anti-rabbit (Alexa-Fluor 430) and goat anti-mouse (Alexa-Fluor 488) were diluted 1:2,000 in 0.5% ELK/PBS and incubated in the dark for 45 minutes. Finally, nuclei were stained with Hoechst (Fisher Scientific; 1:100 in PBS) for 2 minutes. Between each of the above treatments, cells were washed 3 times with PBS. Cells were visualized using a Zeiss Axio Observer Z1 inverted microscope at 40x magnification using an oil-immersion objective. Quantitative image analysis was performed using CellProfiler (v4.2.8). Nuclei were identified based on Hoechst staining, after which a cytoplasmic ring was obtained by expanding the nuclear mask by 30 pixels and subtracting the nucleus. Mean SNRNP70 and dsRNA fluorescence intensities were quantified separately in the nuclear and cytoplasmic compartments. For foci quantification, nuclei were excluded with an additional 5-pixel margin, local background fluorescence was subtracted, and discrete SNRNP70 foci (4-9 pixels in diameter) were identified using a fixed intensity threshold applied across all conditions.

### Northern Blot

Total RNA was isolated from cell monolayers using TRIzol reagent (Invitrogen) following the manufacturer's protocol. Total RNA was quantified using a NanoDrop UV photospectrometer. RNA samples were prepared by mixing 5 µg of total RNA in RNase-free water to a volume of 7 µl, followed by the addition of 7 µl of formamide loading buffer (475 µl of formamide, 15 µl of RNase-free H_2_O, and 10 µl of 0.5 M EDTA [pH 8.0], supplemented with bromophenol blue). Samples were heated at 95°C for 3 minutes to dissociate RNA secondary structures. RNA was separated by denaturing gel electrophoresis in a 6% polyacrylamide gel (4.2 g of Urea, 0.5 ml of 10x TBE [175.3 g/L NaCl, 88.2 g/L sodium citrate, pH 7.0], 1.5 ml of 40% Acrylamide/Bis-acrylamide, 150 µl of APS, and 15 µl of TEMED in a total volume of 10 ml of RNase-free H2O). RNA was semidry blotted onto a Hybond-N membrane (Amersham Biosciences) for 60 minutes at a constant amperage of 0.5 A and UV cross-linked by 1 minute of UV light exposure on each side. DIG-labeled probes targeting the complete sfRNA1 sequence were generated by PCR using the DIG-probe synthesis kit (Roche; Cat#11636090910) and primers: FW, 5′-GCAGTCTGCGATAGTGCC-3′ RV, 5′-GTTGTGCAGAGCAGAAGATC-3′ and pCC1-WNV as a template. The membrane was hybridized with DIG-labeled probes in ExpressHyb Hybridization Solution (Takara Bio) at 68°C. Subsequently, the membrane was washed with 0.1 M maleic acid buffer (0.1 M maleic acid, 0.15 M NaCl, and 0.3% [v/v] Tween-20, pH 7.5), blocked with 0.5% blocking reagent (Roche), and stained with AP-labeled anti-DIG antibodies (Roche) for 30 minutes. The blot was developed using NBT/BCIP substrate (Roche) until the desired signal intensity was reached and scanned using a Bio-Rad Gel Doc scanner. Band intensities were quantified using Image Lab software (Bio-Rad).

### Statistical Analysis

For proteomic data analysis, LFQ values were first log10-transformed, missing values were assigned the lowest log10 (LFQ) value in the dataset, followed by statistical testing using a 2-tailed Student's *t*-test in Perseus (v1.6.15.0) with a permutation-based truncation (FDR 0.05). GO enrichment analysis was performed using PANTHER (v.17) on geneontology.org and STRING (v.12.0), using default settings in the Fisher's exact test and FDR correction for multiple testing. Top 5 significant GO terms are ranked based on their signal, representing a weighted harmonic mean of the observed/expected ratio and -log(FDR) that balances statistical significance with effect size. To identify functional protein modules within the interaction networks, we performed Markov Clustering (MCL) analysis with default parameters to group proteins that showed similar binding profiles to UpA-rich RNA sequences. Analysis of WNV genomic dinucleotide composition for each region was performed using SSE v1.4 (Simmonds, 2012). Statistical analyses of experimental data were performed using GraphPad Prism (v10.2.2). Depending on the experimental design, paired comparisons were analyzed using a paired *t*-test on log-transformed values, comparisons among multiple groups were analyzed using the Kruskal-Wallis test followed by Dunn's multiple-comparisons test, and correlations were assessed using Spearman's rank correlation. The statistical test used for each experiment is indicated in the corresponding figure legends.

## RESULTS

### Virus Infection Increases the Expression of Antiviral Proteins and Alters the Host’s RNA-Binding Proteome

To identify host proteins that specifically interact with UpA-enriched RNA sequences in the context of viral infection, we first investigated how viral infection itself affects the cellular proteome and subsequently may alter RNA-binding protein profiles. Therefore, we performed mass spectrometry analysis on human A549 cells at 24 hours post infection with the orthoflavivirus ZIKV (MOI = 5) to characterize infection-induced proteome changes. ZIKV was selected because UpA dinucleotides are globally suppressed across its RNA genome (observed/expected [O/E] ratio = 0.52), similar to cellular mRNA (average human O/E ratio = 0.55) (Kariin and Burge, 1995). Comparison of infected and uninfected cell lysates revealed upregulation of multiple antiviral factors, including the interferon-induced proteins myxovirus resistance protein 1 (MX1), interferon-induced protein with tetratricopeptide repeat 1 (IFIT1), retinoic acid-inducible gene I [RIG-I; DEAD-box helicase 58 (DDX58)], and protein kinase R (PKR), as well as the immune signaling components signal transducer and activator of transcription 1 (STAT1) and nuclear factor kappa B subunit 2 (NFKB2), and the protein modification enzymes ubiquitin-conjugating enzyme E2 L6 (UBE2L6) and Deltex E3 ubiquitin ligase 3L (DTX3L) (Fig. 1A). GO enrichment analysis confirmed significant over-representation of antiviral defense and innate immune response pathways (Fig. 1B), with STRING analysis revealing that these proteins form an interconnected functional network (Fig. 1C).

**Fig. 1:**
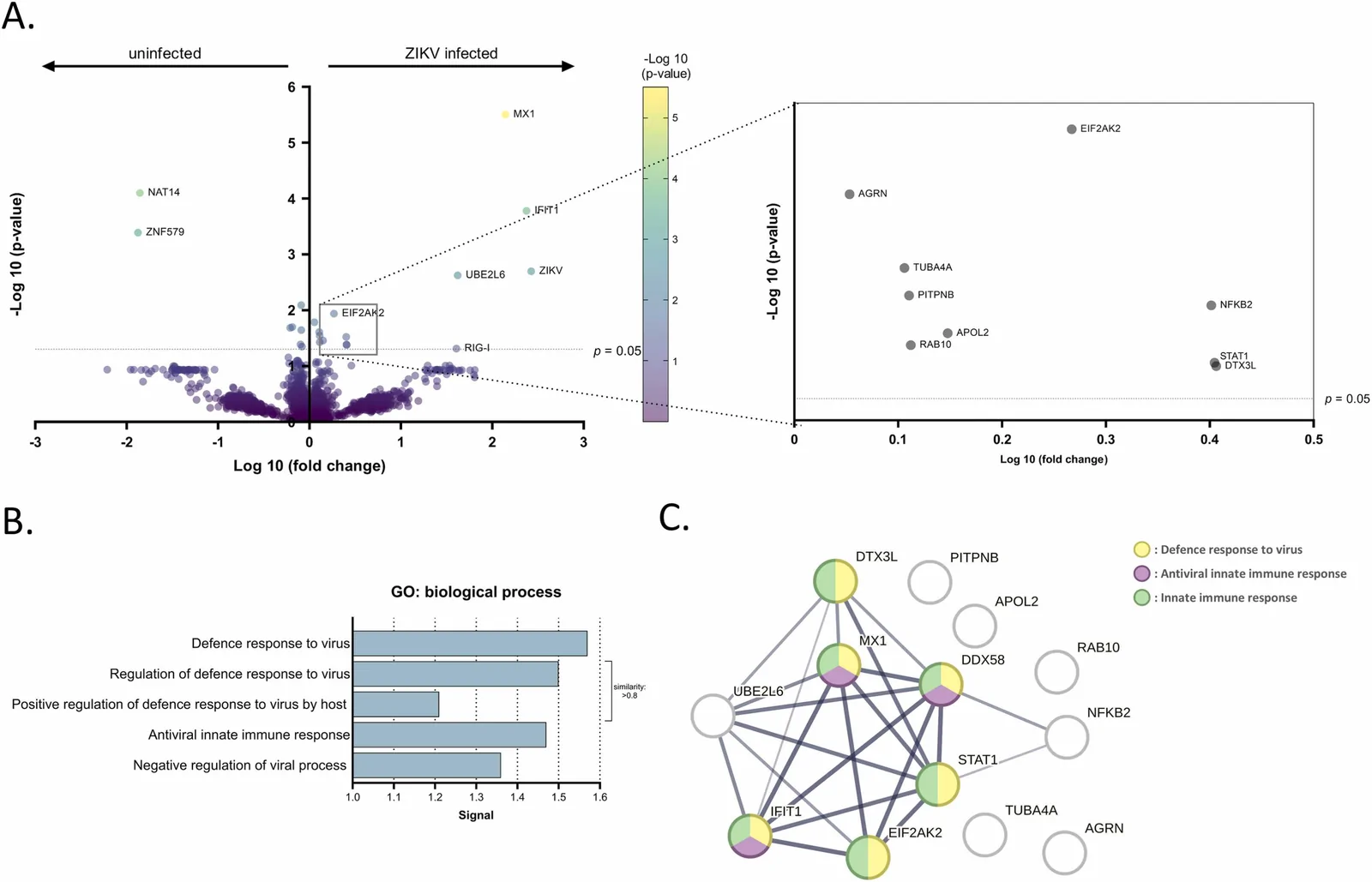
Antiviral proteins expressed by viral infection display differential affinity for UpA-high RNA. (A) Volcano plot showing differential protein expression between Zika virus (ZIKV)-infected and uninfected A549 cells. Data represent results from 3 independent RNA affinity purification experiments. (B) Top enriched Gene Ontology (GO) terms for biological processes in ZIKV-infected cells. (C) STRING protein-protein interaction network of differentially expressed proteins in ZIKV-infected cell lysates.

Next, we examined whether this altered proteome also changes the profile of host proteins that bind to RNA sequences with varying UpA dinucleotide content. We developed an RNA affinity purification approach using lysates from both uninfected and ZIKV-infected cells, combined with RNA baits designed to capture UpA-specific interactions. Three distinct RNA baits were constructed, each lacking transcriptional and translational regulatory elements and fused to streptavidin-binding RNA aptamers for purification (Fig. 2A). The control bait (UpA-low) contained an RNA sequence derived from ZIKV with naturally low UpA content [57 UpAs, (O/E) ratio = 0.52]. To specifically capture UpA-interacting proteins, we engineered a second bait (UpA-high) with identical length and mononucleotide frequencies but containing 150 additional UpA dinucleotides (207 total, O/E ratio = 1.72). A third artificial bait (UpA-low_a) with similar UpA content (49 UpAs, O/E ratio = 0.53) but different sequence arrangement controlled for sequence context effects. Mass spectrometry analysis identified several host proteins with altered RNA-binding profiles during infection (Supplementary Fig. 1). Three factors demonstrated increased binding to all RNA baits when incubated with infected lysates compared with uninfected controls: RNA helicase with zinc-finger 2 (HELZ2), poly(ADP-ribose) polymerase 9 (PARP9), and DTX3L, suggesting infection-induced sequence-independent RNA binding (Fig. 2B). More importantly, 4 proteins demonstrated exclusive enrichment on UpA-high RNA specifically during infection: the antiviral factors IFIT1 and 2′- to 5′-oligoadenylate synthetase 1 (OAS1), along with ubiquitin protein ligase E3B (UBE3B) and peptidyl-prolyl isomerase-like 2 (PPIL2) (Fig. 2B). Notably, both DTX3L and IFIT1 were also among the proteins with increased abundance in ZIKV-infected cell lysates, suggesting that their enhanced RNA binding is indeed infection-induced. Network analysis revealed that most proteins binding UpA-high RNA during infection formed an interconnected antiviral network (Fig. 2C), with GO enrichment confirming their roles in innate immune responses (Fig. 2D). Notably, 2 proteins fell outside this network: PPIL2 and UBE3B, which showed exclusive binding to UpA-high RNA during infection. While UBE3B functions primarily in protein degradation pathways, PPIL2 has dual roles in both ubiquitination and splicing processes (Bai et al., 2021).

**Fig. 2:**
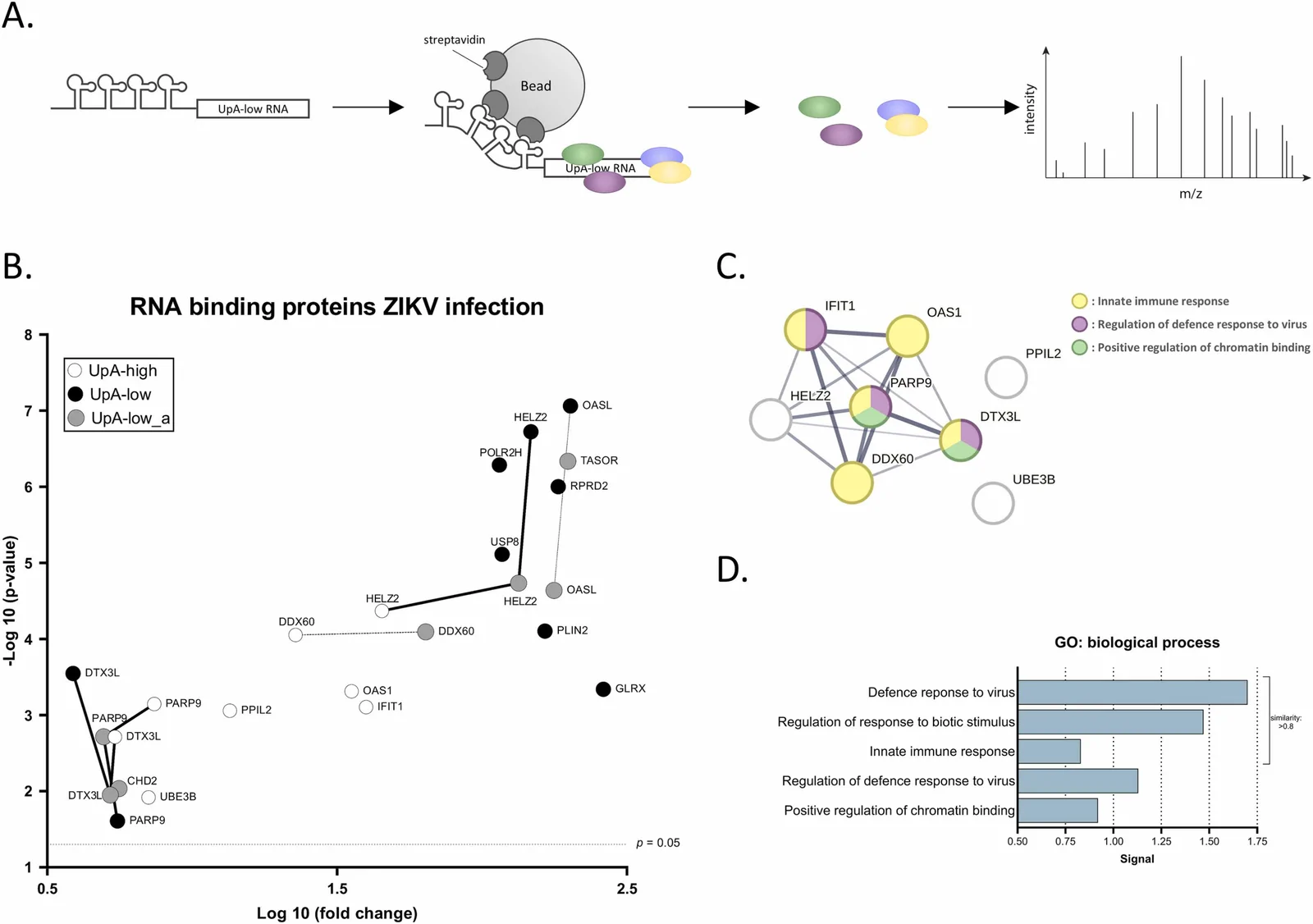
Proteins expressed during viral infection display differential affinity for UpA-high RNA. (A) Schematic representation of the RNA affinity purification assay workflow. (B) Volcano plot showing differential protein binding to UpA-high (white), UpA-low (black), or artificially UpA-low (UpA-low_a; gray) RNA sequences between ZIKV-infected and uninfected A549 cells. Data represent results from 3 independent RNA affinity purification experiments. (C) STRING protein-protein interaction network of proteins showing preferential binding to UpA-high RNA during infection. (D) Top enriched GO terms for biological processes in UpA-high RNA-binding protein set.

### Proteins Involved in Early Splicing Bind With Higher Affinity to Sequences Rich in UpA Dinucleotides

Given that all organisms display suppressed UpA dinucleotide frequencies in cellular RNA (Karlin and Burge, 1995), we next sought to identify host proteins that intrinsically prefer UpA-enriched sequences regardless of infection status. To identify proteins that preferentially interact with UpA-enriched RNA sequences, we directly compared proteins that showed strong affinity for UpA-high RNA with those that associated with UpA-low RNAs, using cell lysates from both ZIKV-infected and uninfected A549 cells (Supplementary Fig. 2). However, only a small number of proteins showed significant differences in each comparison with limited overlap between experimental conditions, which precluded meaningful network and GO enrichment analyses.

Because only a limited number of proteins reached statistical significance in the direct comparison between the artificial RNA baits, we next performed an exploratory analysis to identify broader trends in UpA-dependent RNA binding. For this purpose, proteins displaying at least 2 fold greater binding to UpA-high RNA than to both UpA-low controls were included. This more inclusive approach revealed 283 and 234 proteins that preferentially bound to UpA-high RNA using ZIKV-infected and uninfected cell lysates, respectively (Fig. 3A). GO enrichment analysis showed minimal differences in GO terms between both conditions and highlighted an enrichment for RNA-binding proteins, particularly those involved in various RNA metabolic processes (Supplementary Fig. 3).

**Fig. 3:**
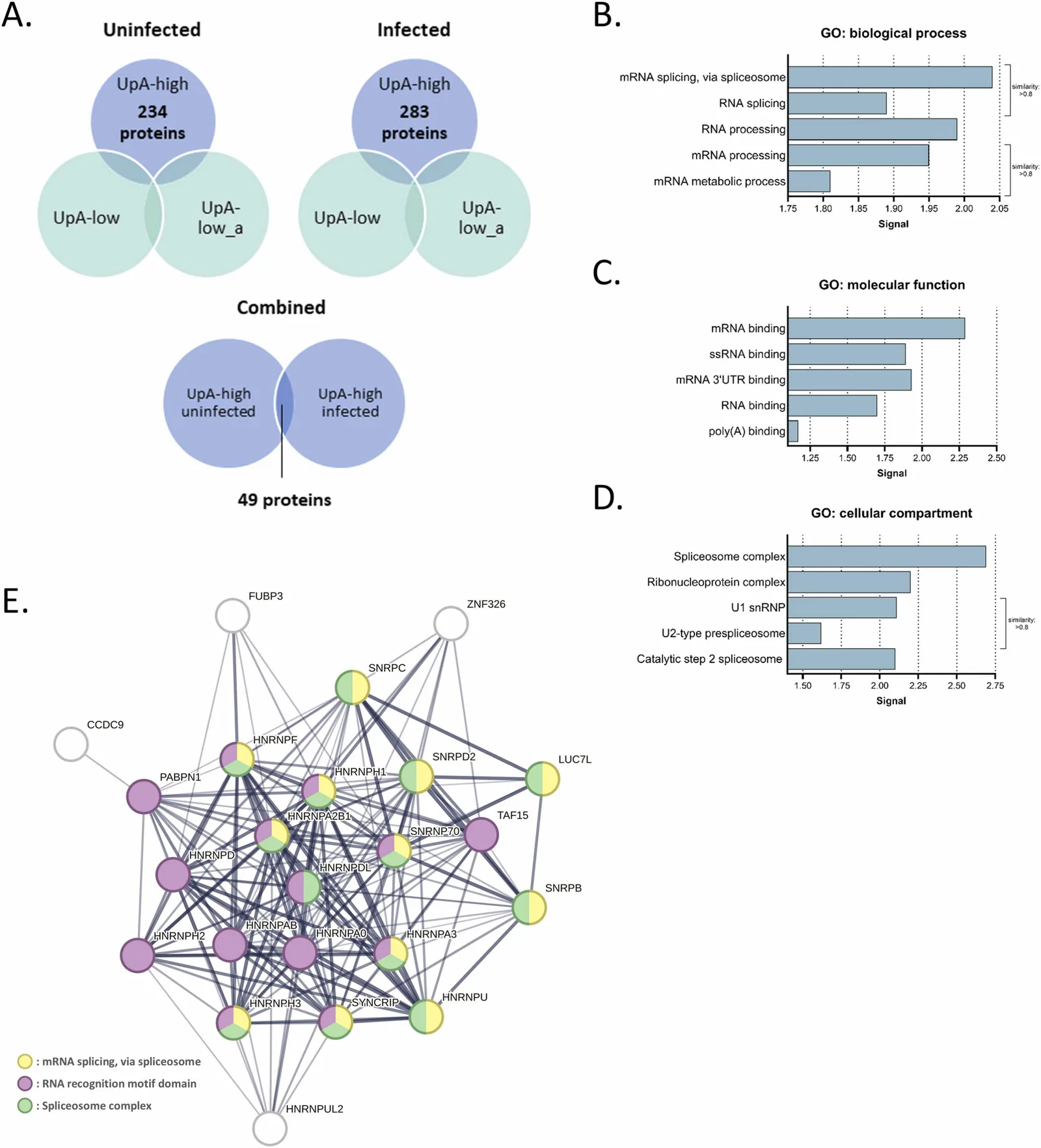
Artificial RNA sequences high in UpA dinucleotides preferentially recruit RNA-processing and splicing factors independent of infection status. (A) Venn diagrams showing the number of proteins with increased binding to UpA-high compared with both UpA-low and UpA-low artificial (UpA-low_a) RNA in uninfected and Zika virus (ZIKV)-infected conditions. Data represent results from 3 independent RNA affinity purification experiments. (B-D) Top enriched gene ontology (GO) terms of the 49 proteins that consistently bind UpA-high RNA. Analysis includes biological process, molecular function, and cellular compartment categories. (E) STRING protein-protein interaction network of the core cluster of the 49 proteins showing enhanced binding to UpA-high RNA compared with UpA-low and UpA-low_a sequences in both uninfected and infected conditions.

Comparison between the 2 datasets revealed 49 proteins that consistently bound to UpA-high RNA sequences in both infected and uninfected conditions (Fig. 3A). GO enrichment analysis of this protein set identified enrichment in biological processes related to mRNA splicing via spliceosomes (Fig. 3B). Molecular function analysis highlighted enrichment in ssRNA binding, poly(A) interaction, and mRNA 3'-UTR recognition capabilities (Fig. 3C). Cellular compartment analysis demonstrated association with U1 small nuclear ribonucleoprotein (snRNP) and U2-type prespliceosome complexes (Fig. 3D). Importantly, reciprocal analysis of proteins preferentially binding to natural and artificial UpA-low sequences showed no enrichment for splicing-related GO terms, indicating a specific association between elevated UpA content and splicing factor recruitment (Supplementary Fig. 4). Network analysis of the UpA-high binding proteins (Supplementary Fig. 5) identified one large, interconnected cluster enriched for RNA processing and spliceosomal mRNA splicing activities (Fig. 3E). Collectively, these results strongly suggest that early components of the splicing machinery preferentially recognize and interact with RNA sequences containing elevated UpA dinucleotide frequencies.

To investigate whether spliceosomal components also exhibit affinity for naturally occurring UpA-rich RNA, we next examined WNV, a closely related flavivirus. WNV exhibits genome-wide UpA suppression (O/E ratio = 0.49) similar to ZIKV. However, in contrast to ZIKV, WNV differs markedly in its 3′ UTR, which contains a notably high UpA ratio (O/E = 0.89) (Fig. 4A). Detailed examination of the WNV 3’ UTR identified 3 distinct UpA-enriched regions: (1) the first 150 nucleotides containing stem-loop (SL) I and part of SLII, (2) nucleotides 300 to 450 from SL3 up to and including dumbbell (DB) I, and the terminal 100 nucleotides known as the 3′SL (Fig. 4B). These RNA structures form the 3’-UTR exoribonuclease-resistant RNAs (xrRNAs) that function as 5′- to 3′-exoribonuclease XRN1 stalling sites (Fig. 4B). Stalling of XRN1 results in the production of subgenomic flavivirus RNA (sfRNA) (Chapman et al., 2014; Funk et al., 2010). RNA affinity purification, using the complete WNV 3′ UTR and a natural UpA-low sequence coding for WNV NS2A, identified multiple proteins that showed significant preference (*p* < 0.05) for the 3′ UTR over the NS2A region (Supplementary Fig. 6). These proteins formed a highly interconnected network (Supplementary Fig. 7) of which the core network (Figure 4C) strikingly resembled our previous findings with artificial UpA-enriched sequences (Fig. 3E). GO enrichment analysis revealed that the dataset of identified 3′-UTR-interacting proteins is significantly enriched for mRNA processing factors (Fig. 4D) and for AU-rich mRNA 3’-UTR-binding proteins (Figure 4E), with splicing components being particularly prominent (Fig. 4F). In contrast, the reciprocal analysis of proteins preferentially binding to the NS2A sequences showed no enrichment for splicing-related GO terms, indicating a specific association between WNV 3’ UTR and splicing factors (Supplementary Fig. 8). Proteins identified with artificially enriched and naturally occurring UpA-high RNAs are detailed in Table 1. Notably, almost all overlapping proteins are known ARE-binding factors, with some displaying clear binding motifs containing one or multiple UpA dinucleotides, and many having established roles in mRNA splicing (Table 1).

**Fig. 4:**
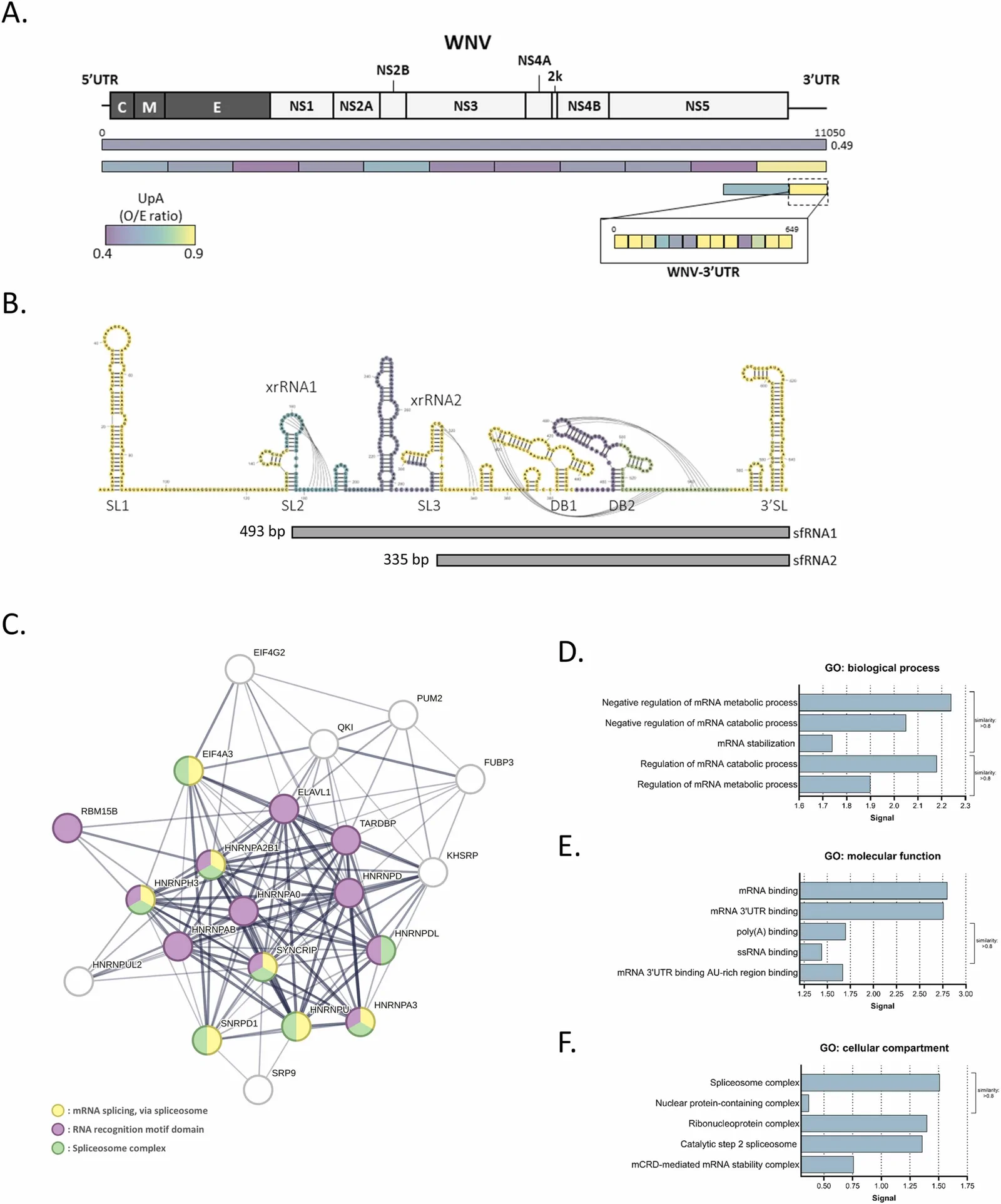
Natural RNA sequences in the 3’ UTR of West Nile virus are high in UpA dinucleotides and recruit RNA-processing and splicing factors. (A) Genome-wide UpA dinucleotide suppression in West Nile virus (WNV), highlighting the distinct UpA ratio in the WNV 3′ UTR. A rolling window approach with a 1000-nucleotide window for the whole genome and a 50-nt window for the WNV 3’ UTR was used to analyze different genomic regions. The color code represents the observed-over-expected (O/E) ratio for each genomic region. (B) Detailed RNA structure of the WNV 3’ UTR with indicated stem-loop (SL) and dumbbell (DB) structures, and exoribonuclease (XRN) stalling sites (xrRNA). Color codes representing the O/E UpA dinucleotide ratio. Created using RNAcanvas. (C) STRING protein-protein interaction network of proteins significantly interacting with the WNV 3′-UTR RNA compared with the control (NS2A) RNA. (D-F) Top enriched gene ontology (GO) terms of the proteins that bind to the WNV 3’ UTR. Analysis includes biological process (D), molecular function (E), and cellular compartment (F) categories.

**Table 1:** Proteins binding to artificial and naturally occurring UpA-high RNA sequences.

Gene	Protein	RNA binding	Binding motif	Description	Ref.
hnRNPD	Heterogeneous nuclear ribonucleoprotein D0	mRNA 3’-UTR AU-rich elements	5’-UUAG-3’	Involved in translationally coupled RNA turnover. Key factor broadly involved in cytoplasmic mRNA turnover via ARE-mediated mRNA decay and *fos* protein-coding region determinant of instability	Xu et al., 2001
hnRNPA0	Heterogeneous nuclear ribonucleoprotein A0	AU-rich elements' mRNA	*n/a*	Splicing regulatory protein binding to exon/intron splicing enhancer or silencer elements. Involved in post-transcriptional regulation of cytokine mRNAs	Rousseau et al., 2002
hnRNPDL	Heterogeneous nuclear ribonucleoprotein D-like	mRNA 3’- UTR AU-rich elements	5’-ACUAGC-3’	Binds with high affinity to AREs found within 3’ UTR of many proto-oncogenes and cytokines' mRNA. Regulates transcription and alternative splicing	Li et al., 2019
hnRNPH3	Heterogeneous nuclear ribonucleoprotein H3	RNA binding	*n/a*	Involved in regulating the mRNA splicing via spliceosome process and participates in early heat shock-induced splicing arrest	Mahé et al., 1997
hnRNPUL2	Heterogeneous nuclear ribonucleoprotein U like 2	RNA binding	*n/a*	Predicted to be involved in mRNA splicing via spliceosome	
hnRNPA3	Heterogeneous nuclear ribonucleoprotein A3	RNA binding	*Cis*-acting response element A2RE	Plays a role in cytoplasmic trafficking of RNA. Involved in RNA binding, mRNA transport, and splicing. Part of catalytic step 2 spliceosome	Chen et al., 2025
hnRNPAB	Heterogeneous nuclear ribonucleoprotein A/B	ssRNA G-rich and U-rich	*n/a*	Also binds to APOB mRNA transcripts around the RNA editing site. Stimulates alternative splicing of several mammalian and viral pre-mRNAs	Martinez-Contreras et al., 2006
hnRNPA2B1	Heterogeneous nuclear ribonucleoproteins A2/B1	pre-mRNA, miRNA	*Cis*-acting response element A2RE (Burns et al., 2009)	Plays a role in processes such as transcription, pre-mRNA processing, RNA nuclear export, subcellular location, mRNA translation, and stability of mature mRNAs. Also plays a role in activation of innate immune response via activation of the TBK1-IRF3 pathway	Alarcón et al., 2015; Akiyama et al., 2016
PUM2	Pumilio homolog 2	mRNA 3’ UTR	5’-UGUANAUA-3’	Binds the Pumilio response element (PRE) and acts as post-transcriptional repressor via various mechanisms; mRNA degradation, translational inhibition, and enhancing miRNA regulation. Pumilio also functions in cytoplasmic viral sensing	Goldstrohm et al., 2018, Lu and Hall, 2011, Van Etten et al., 2012
hnRNPU	Heterogeneous nuclear ribonucleoprotein U	RNA binding	*n/a*	Involved in cellular processes such as nuclear chromatin organization, telomere-length regulation, transcription, mRNA alternative splicing, and stability by regulating U2 snRNP biogenesis, Xist-mediated transcriptional silencing, and mitotic cell progression	Xiao et al., 2012, Fackelmayer et al., 1994
SYNCRIP	Heterogeneous nuclear ribonucleoprotein Q	AU-rich RNA, 5’ and 3’ UTR	5’-UCUAA-3’	Component of the CRD-mediated complex that promotes MYC mRNA stability. Isoform 1, isoform 2, and isoform 3 are associated *in vitro* with pre-mRNA, splicing intermediates, and mature mRNA protein complexes	Choi et al., 2004
FAM120A	Constitutive coactivator of PPAR-gamma-like protein 1	RNA binding	*n/a*	Functions as a scaffolding protein in oxidative stress-induced survival signaling, regulating SRC family kinase activation and PI3-kinase phosphorylation, while also binding IGF2 RNA to promote IGF2 protein production	Kelly et al., 2019
FUBP3	Far upstream element binding protein 3	*n/a*	*n/a*	Involved in DNA-templated transcription; positive regulation of DNA-template transcription; and positive regulation of gene expression. Known to regulate HIV-1 transcription and immune response pathway in T cells	Marichannegowda et al., 2025

Previously described RNA-binding capabilities are mentioned in the “RNA binding” column.

Proteins were identified by mass spectrometry analysis using human A549 cell lysates. Functions were assessed using the UniProt database annotations.

### Early Spliceosome Components Shuttle to the Cytoplasm During Infection and Contribute to UpA-Dependent Flavivirus Restriction

Pre-mRNA splicing is an essential nuclear RNA-processing mechanism in which introns are removed from precursor mRNAs by the spliceosome (Figure 5A). Since our RNA pull-down analyses identified multiple early spliceosome components, we focused on the small nuclear ribonucleoprotein U1 subunit 70 (SNRNP70), a core component of the first stage of spliceosome assembly. Because flaviviruses exclusively replicate in the cytoplasm whereas pre-mRNA splicing occurs in the nucleus, we first examined the subcellular localization of SNRNP70 during infection. In uninfected Vero cells, SNRNP70 displayed a strong nuclear localization. In contrast, infection with either WNV or ZIKV resulted in a redistribution of SNRNP70 to the cytoplasm at 24 hours post infection, where SNRNP70 was mostly observed in cytoplasmic foci (MOI = 10) (Figure 5B). Cytoplasmic SNRNP70 abundance positively correlated with intracellular dsRNA levels on a single-cell basis in both WNV- and ZIKV-infected cells (Figure 5C,D), indicating that SNRNP70 relocalization is associated with viral RNA accumulation. However, SNRNP70 did not visibly colocalize with dsRNA-positive replication complexes (Figure 5B). Consistent with these observations, quantitative image analysis demonstrated a significant increase in the cytoplasmic-to-nuclear SNRNP70 intensity ratio following ZIKV infection (Figure 5E). Similarly, quantification of discrete cytoplasmic SNRNP70 foci showed clear foci formation, particularly following ZIKV infection (Figure 5F).

**Fig. 5:**
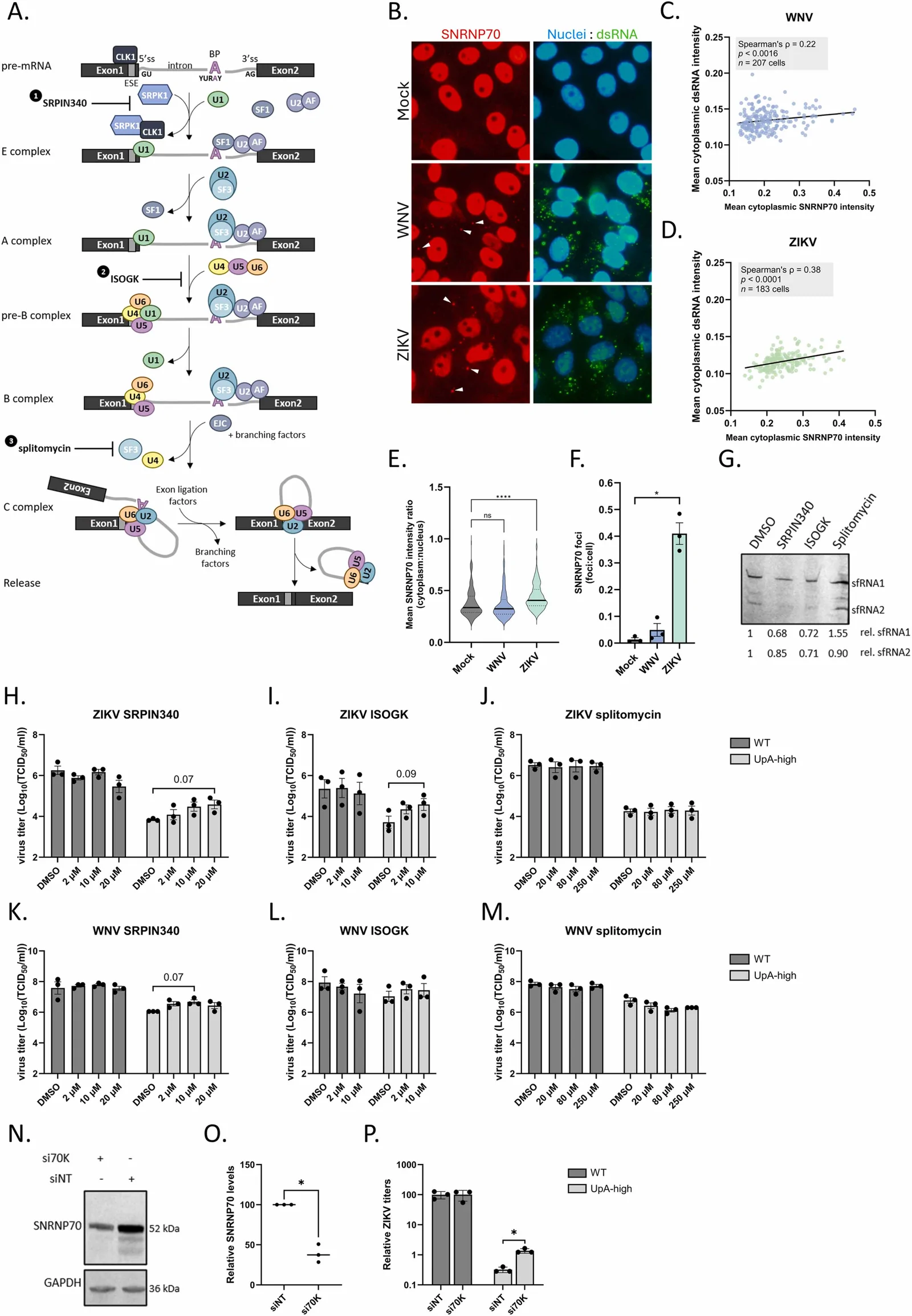
*Components of the early spliceosome affect replication and sfRNA formation of UpA-high flavivirus RNA.* (A) Schematic overview of the splicing pathway. Indicated are points of intervention for the 3 inhibitors used in this study: SRPIN340 (inhibits U1 binding), ISOGK (prevents complex B formation), and splitomycin (blocks splicing after spliceosome assembly). (B) Immunofluorescence analysis of SNRNP70 localization in uninfected (mock) and West Nile virus (WNV)- and Zika virus (ZIKV)-infected Vero cells. White arrows indicate cells with cytoplasmic SNRNP70 localization. (C) Correlation between mean cytoplasmic SNRNP70 and dsRNA intensities in WNV-infected cells. (D) Correlation between mean cytoplasmic SNRNP70 and dsRNA intensities in ZIKV-infected cells. (E) Quantification of the cytoplasmic-to-nuclear SNRNP70 intensity ratio in mock-, WNV-, and ZIKV-infected cells. (F) Quantification of discrete cytoplasmic SNRNP70 foci in mock-, WNV-, and ZIKV-infected cells at 24 hours post infection. (G) Northern blot analysis of subgenomic flavivirus (sf)RNA production of WNV following treatment with the various splicing pathway inhibitors. Numbers represent the average signal intensities for sfRNA1 and sfRNA2 from independent experiments. Individual Northern blots provided as Supplementary Figure 10. (H-J) Viral titers of wild-type (WT) and UpA-high ZIKV following treatment with SRPIN340, ISOGK, or splitomycin, respectively. (K-M) Viral titers of WT and UpA-high West Nile virus (WNV) following treatment with SRPIN340, ISOGK, or splitomycin, respectively. (N) Western blot analysis confirming siRNA-mediated depletion of SNRNP70. (O) Quantification of relative SNRNP70 protein levels following siRNA-mediated knockdown. (P) Relative titers of WT and UpA-high ZIKV following SNRNP70 knockdown. Data are presented as mean ± SEM from 3 independent experiments unless otherwise indicated. Differences in cytoplasmic-to-nuclear SNRNP70 fluorescence intensity ratios were analyzed using the Kruskal-Wallis test followed by Dunn's multiple-comparisons test. Associations between cytoplasmic SNRNP70 and dsRNA fluorescence intensities were analyzed using Spearman's rank correlation. Differences in virus titers between DMSO-treated cells and the indicated inhibitor concentrations were analyzed using the Kruskal-Wallis test followed by Dunn's multiple-comparisons test. Differences in relative UpA-high ZIKV titers following SNRNP70 knockdown were analyzed using a paired t-test on log-transformed values. (* *p* < 0.05) was considered statistically significant.

Although the RNA affinity purification experiments were performed using whole-cell lysates, which do not preserve intracellular compartmentalization, the infection-induced cytoplasmic relocalization of SNRNP70 suggests that at least a subset of the identified interactions may occur under physiological conditions. To determine whether early spliceosome components functionally contribute to flavivirus replication, we employed 3 molecular inhibitors targeting distinct stages of the splicing pathway: (1) SRPIN340, which blocks presplicing events by inhibiting SRPK-CLK1 complex formation and subsequent U1 binding (Aubol et al., 2016); (2) ISOGK, which stalls complex A by preventing complex B formation by blocking U4/U5/U6 tri-snRNP recruitment (Effenberger et al., 2016; Murphy et al., 2022); and (3) splitomycin, which inhibits the splicing reaction after complete spliceosome assembly (Kuhn et al., 2009) (Fig. 5B). Noncytotoxic concentrations were determined, and the splicing inhibition efficacy was verified using GAPDH exon junction formation analysis (Supplementary Fig. 9).

We first examined how pharmacological inhibition of splicing may affect XRN1 stalling and sfRNA production from the naturally UpA-high WNV 3’ UTR. WNV produces at least 2 sfRNAs that play critical roles in replication during flavivirus infection (Akiyama et al., 2016; Bustos-Arriaga et al., 2018; Doets and Pijlman, 2024; Pijlman et al., 2008; Roby et al., 2014; Zhang et al., 2020). Northern blot analysis revealed that splicing inhibitors SRPIN340 and ISOGK decreased sfRNA levels, while splitomycin treatment increased the levels of sfRNA1 and sfRNA2 (Fig. 5G). This suggests that early spliceosome components may contribute to efficient sfRNA accumulation during WNV infection.

Synonymous introduction of UpA dinucleotides into viral genomes typically attenuates replication (Atkinson et al., 2014; Burns et al., 2009; Fros et al., 2017; Fros et al., 2021; Tulloch et al., 2014). Therefore, we assessed whether inhibition of the spliceosome differentially affects the replication of wild-type (UpA-low) or synonymously recoded UpA-high flaviviruses. Vero cells were pretreated with different concentrations of each inhibitor and 2 hours later infected with either wild-type (WT) or UpA-high mutant ZIKV or WNV (MOI = 0.1). SRPIN340 treatment showed a dose-dependent trend toward enhanced replication of the UpA-high ZIKV (Fig. 5H). Similarly, ISOGK treatment was associated with a concentration-dependent increase in UpA-high virus replication, particularly for ZIKV (Fig. 5I). However, these effects did not reach statistical significance (*p* = 0.07 and 0.09, respectively). In contrast, splitomycin treatment had no effect on ZIKV replication across all concentrations tested (Fig. 5J). Similarly, a UpA-high WNV mutant displayed a trend toward rescued replication by inhibition of the early steps in the splicing pathway (Fig. 5K,L) and not when treated with an inhibitor that functions after complete spliceosome assembly (Fig. 5M).

As SNRNP70 cytoplasmic relocalization and the effects of spliceosome inhibition were more pronounced during ZIKV than WNV infection, subsequent functional studies were performed using ZIKV. To investigate whether SNRNP70 contributes to UpA-dependent restriction, SNRNP70 expression was depleted by siRNA-mediated knockdown prior to infection. Western blot analysis confirmed efficient depletion of SNRNP70 (Fig. 5N), which reduced SNRNP70 protein levels by approximately 60% relative to cells transfected with a nontargeting siRNA (Fig. 5O). SNRNP70 depletion had no effect on WT ZIKV replication, whereas replication of the UpA-high virus increased significantly following knockdown (Fig. 5P). Together, these results demonstrate that early steps in the splicing pathway specifically restrict UpA-high flavivirus replication.

## DISCUSSION

UpA dinucleotide suppression represents one of the most conserved features of RNA composition, observed across virtually all living organisms (Beutler et al., 1989, Burge et al., 1992, Kariin and Burge, 1995, Nussinov, 1984) and mirrored in most viral RNA genomes (Rima and McFerran, 1997; Simmonds et al., 2013). While artificially increasing viral genomic UpA content consistently leads to virus attenuation (Atkinson et al., 2014; Burns et al., 2009; Fros et al., 2017; Fros et al., 2021; Tulloch et al., 2014) and UpA-rich RNA is known to have a higher turnover (Duan and Antezana, 2003), the molecular mechanisms underlying the phenomenon have remained elusive.

Analysis of RNA-protein interactions during ZIKV infection identified 4 proteins with enhanced binding to UpA-high sequences: IFIT1, OAS1, UBE3B, and PPIL2. Notably, none of these proteins were detected in pulldowns using uninfected cell lysates, indicating that their recruitment is infection-dependent. IFIT1 and OAS1 are well-established antiviral effectors, although neither has previously been linked directly to recognition of UpA-rich single-stranded RNA. IFIT1 restricts viruses by binding to the viral RNAs lacking 2′-O methylation at the 5′ cap or containing uncapped 5′-triphosphate termini (Diamond, 2014), whereas OAS1 recognizes dsRNA with a U-/A-rich consensus sequence (5’-NNWWN_9_WG-3’, where N represents any nucleotide and W either A or U) (Kodym et al., 2009) and activates the RNase L pathway (Melchjorsen et al., 2009). In addition, OAS1 protects against WNV infection by binding AREs within specific host mRNAs, including IFN-β, thereby stabilizing these transcripts and prolonging their half-life (Harioudh et al., 2024).

Identification of UBE3B and PPIL2 suggests that the response to UpA-high RNA may additionally involve ubiquitin-dependent regulation of RNA-protein complexes. While both UBE3B possess E3 ubiquitin ligase activity, PPIL additionally functions as a component of the minor spliceosome, where it stabilizes the U5 snRNP through direct interaction with loop I of the U5 snRNA (Bai et al., 2021). PPIL2 may therefore link spliceosomal RNA recognition to ubiquitin-dependent assembly, remodeling, or turnover of ribonucleoprotein complexes. Together, these infection-specific interactors indicate that the fate of UpA-high RNA is unlikely to be determined by a single recognition factor but rather by a heterogeneous RNA-protein environment comprising antiviral effectors, RNA-processing factors, and proteins involved in ubiquitin-dependent regulation. Whether these interactors promote or restrict viral replication is likely to depend on the individual protein and the context in which the interaction occurs.

Consistent with this broader interaction landscape, comparison of the complete protein profiles associated with UpA-high and UpA-low RNA revealed a pronounced enrichment of splicing-associated factors, particularly small nuclear ribonucleoproteins (snRNPs) and heterogeneous nuclear ribonucleoproteins (hnRNPs). snRNPs are RNA-protein complexes that assemble to form the catalytic core of the spliceosome (Buratti, 2013), whereas hnRNPs constitute a diverse family of RNA-binding proteins involved in multiple aspects of RNA metabolism, including pre-mRNA splicing, transcriptional regulation, mRNA transport, stability, and translation (Geuens and Bouhy, 2016, Li et al., 2023).

To assess whether spliceosomal components contribute functionally to UpA-mediated attenuation, we inhibited distinct stages of the splicing pathway and depleted SNRNP70, a core component of the U1 snRNP. Inhibition of early spliceosomal events with SRPIN340 or ISOGK reduced sfRNA accumulation during WNV infection, but this partial reduction did not measurably affect virus replication, suggesting that residual sfRNA was sufficient to support replication. Instead, the predominant phenotype was a dose-dependent rescue of UpA-high ZIKV and WNV replication, whereas inhibition of later splicing events with splitomycin had no effect. Likewise, SNRNP70 depletion enhanced replication of UpA-high ZIKV while having little effect on the corresponding wild-type virus. Together, these findings demonstrate that components involved in early spliceosome assembly, rather than completion of canonical splicing, contribute to the restriction of UpA-high viruses. Although SNRNP70 knockdown only partially rescued replication, likely reflecting residual protein expression and functional redundancy among spliceosomal factors, the selective effect on the UpA-high virus supports a model in which SNRNP70 acts as part of a broader early spliceosomal network regulating the fate of UpA-rich viral RNA. Although pharmacological inhibitors may exert indirect or off-target effects, the concordance between 2 mechanistically distinct early-stage inhibitors and the independent genetic validation by SNRNP70 depletion argues against a purely compound-specific mechanism.

Previous observations provide additional support for noncanonical antiviral functions of spliceosomal components. Depletion of the core U2 snRNP component SF3B1, or treatment with the SF3B1 inhibitor pladienolide B, enhanced replication of several RNA viruses, including Sindbis virus (SINV), Semliki Forest virus, coxsackievirus B3, and severe acute respiratory syndrome coronavirus 2 (SARS-CoV-2) (Bojkova et al., 2020; Kamel et al., 2024; Sun, 2020). These viruses have relatively high genomic UpA observed-to-expected ratios of 0.80, 0.81, 0.83, and 0.83, respectively. Although this relationship does not establish UpA content as the sole determinant of spliceosomal restriction, it is consistent with the possibility that A/U-rich viral RNA features contribute to the recruitment of RNA-processing factors. Furthermore, the cytoplasmic relocalization of SNRNP70 during ZIKV and WNV infection parallels observations made during SINV infection, in which U2 snRNA directly interacts with viral RNA in the cytoplasm (Kamel et al., 2024, Zheng et al., 2023). Direct association of snRNAs with viral genomes has also been documented during SARS-CoV-2 and ZIKV infection (Zhao et al., 2024; Ziv et al., 2018). Collectively, these studies support an emerging model in which spliceosomal components can be repurposed outside the nucleus to interact with cytoplasmic viral RNA.

U1 and U2 snRNAs are the core components of their respective snRNPs and mediate splice-site recognition through sequence-specific RNA-RNA interactions (Black et al., 1985). The U1 snRNA (3’-UC/CAUUCA-5’) base-pairs with pre-mRNA 5’ splice sites (5’-AG/GURAGU-3’, where R represents A or G) (Roca et al., 2013; Rosbash and Séraphin, 1991), while the U2 snRNA base-pairs with the branch point sequence (5′-YURAY-3′, where Y represents C or U) (Bai and Wan, 2021, Black et al., 1985). Beyond these core splicing signals, introns contain AU-rich pentamers that help define intron-exon boundaries (Bakheet et al., 2018, Lim and Burge, 2001). Collectively, these splicing motifs are notably rich in A and U nucleotides, consistent with the observation that introns exhibit higher UpA dinucleotide ratio than exons (Kariin and Burge, 1995). Given this relationship between UpA content and splicing recognition, we propose that viral suppression of UpA dinucleotides may reduce the association of splicing factors with viral RNA and thereby limit their potential restrictive effects on virus replication, possibly through aberrant RNA processing. Supporting this hypothesis, *in silico* analysis demonstrated that alphavirus and picornavirus genomes, both relatively high in genomic UpA dinucleotides, contain fewer branch point sequences required for proper splicing compared with human RNA (Kamel et al., 2024, Zhang et al., 2021).

Analysis of the sequences used in this study revealed distinct splicing motif distributions between naturally occurring and artificially constructed UpA-rich regions. The naturally UpA-high WNV 3′ UTR contained nearly twice as many canonical branch point sequences (YURAY) compared with the UpA-low NS2A region (15 vs 8), whereas in the artificially UpA-enriched sequences, YURAY motifs increased only marginally (from 15 to 18). This differential distribution of splicing recognition motifs provides a compelling explanation for our experimental observations: the natural WNV 3′ UTR showed substantially stronger enrichment of mRNA processing and splicing factors compared with the artificial UpA-high ZIKV constructs. The natural evolutionary selection in the WNV 3′ UTR likely optimized specific motif arrangements for splicing factor interactions, a refinement the artificial recoding strategy did not fully replicate. Despite a limited increase in YURAY motifs, when expressed in the context of an infectious UpA-enriched ZIKV, virus replication was successfully attenuated (Fros et al., 2021), suggesting that additional antiviral factors beyond splicing components may contribute to virus attenuation. Herein lies the hypothesis that UpA dinucleotides serve as central components in multiple RNA-protein-binding sites engaging various host restriction factors next to splicing components that affect RNA fate, including IFIT1 and OAS1, as evident in our mass spectrometry analysis.

Together, our findings reveal that UpA-high RNA sequences associate with various proteins, including early splicing components, uncovering a noncanonical cytoplasmic role for these predominantly nuclear RNA-binding proteins in restricting viral RNAs with elevated UpA content. This dual functionality may contribute to the evolutionary pressure to suppress UpA dinucleotides across most viral RNA genomes and cellular mRNAs, while also providing a potential explanation for how some viruses strategically maintain UpA-rich regulatory regions despite this pressure, as observed in the 3’ UTR of WNV, where splicing components appeared to positively affect sfRNA production. While the initial RNA affinity purification experiments were performed in whole-cell lysates and therefore cannot exclude nonphysiological RNA-protein interactions, the infection-induced cytoplasmic relocalization of SNRNP70, together with the pharmacological and genetic validation experiments, supports the physiological relevance of an antiviral role for early spliceosome components during flavivirus infection. Given that the spliceosome represents one of nature's most ancient and conserved machineries, evolving from bacterial introns during early eukaryotic evolution (Schmitz-Linneweber et al., 2015), our findings raise the possibility that the association of splicing factors with UpA-rich RNA and their restrictive effects on UpA-high viruses contribute to the widespread evolutionary suppression of UpA dinucleotides.

## CRediT authorship contribution statement

**Joyce W. M. van Bree:** Writing - review and editing, writing - original draft, methodology, investigation, formal analysis, data curation, and conceptualization. **Kristel Doets:** Writing - review and editing, investigation, formal analysis, and data curation. **Carmen van de Waterbeemd:** Writing - review and editing, investigation, formal analysis, and data curation. **Sjef Boeren:** Writing - review and editing, methodology, and data curation. **Monique M. van Oers:** Writing - review and editing, supervision, and funding acquisition. **Gorben P. Pijlman:** Writing - review and editing, supervision, and funding acquisition. **Jelke J. Fros:** Writing - review and editing, writing - original draft, supervision, resources, project administration, funding acquisition, formal analysis, and conceptualization.

## Declaration of Competing Interests

The authors declare that they have no known competing financial interests or personal relationships that could have appeared to influence the work reported in this paper.

## Footnotes

Appendix A Supplementary data associated with this article can be found in the online version at doi:10.1016/j.mocell.2026.100404.
