# Supplementary material for: Presplicing factors associate with UpA-rich RNA and limit flavivirus replication
Source: Mol Cells. 2026 Oct 5;49(12):100404. doi: 10.1016/j.mocell.2026.100404 (PMC13737400; doi:10.1016/j.mocell.2026.100404)
Supplement: Supplementary file 2 [file mmc3.docx]

**Supplementary Figure 1. Analysis of infection-induced changes in RNA-protein interactions.** Volcano plots comparing protein binding profiles between Zika virus infected and uninfected conditions for (A) UpA-low, (B) artificial UpA-low (UpA-low_a), and (C) UpA-high RNA sequences. The x-axis shows log10 fold change in protein abundance (infected/uninfected), and the y-axis shows the -log10 of the *p*-value. Statistical significance was determined using a two-tailed Student's t-test. Proteins with significant changes in expression (*p* < 0.05) are shown above the dotted line. Data represent results from three independent RNA affinity purification experiments.

**Supplementary Figure 2. UpA-dependent RNA-protein interactions in uninfected and Zika virus infected cells.** Volcano plots comparing protein binding profiles between (A) UpA-high and UpA-low, and (B) UpA-high and artificial UpA-low (UpA-low_a) sequences using non-infected cell lysates; and (C) UpA-high and UpA-low, and (B) UpA-high and UpA-low_a sequences using Zika virus infected cell lysates. The x-axis shows log10 fold change in protein abundance (infected/uninfected), and the y-axis shows the -log10 of the *p*-value. Statistical significance was determined using a two-tailed Student's t-test. Proteins with significant changes in expression (*p* < 0.05) are shown above the dotted line. Data represent results from three independent RNA affinity purification experiments.

**Supplementary Figure 3. Gene ontology analysis reveals enrichment of RNA processing factors binding to UpA-high sequences.** Gene ontology (GO) enrichment analysis of proteins showing higher affinity for UpA-high RNA compared to both UpA-low and artificial UpA-low (UpA-low_a) sequences in (A) uninfected and (B) Zika virus (ZIKV)-infected A549 cell lysates. Analysis includes biological process, molecular function, and cellular compartment categories. Terms are plotted according to their signal strength (x-axis), which combines the enrichment effect size (observed/expected ratio) and statistical significance (-log10 FDR). Circle size indicates the number of proteins in each term. GO terms with more than 80% semantic similarity are clustered and shown in the same colour to reduce redundancy.

**Supplementary Figure 4. Analysis of proteins preferentially binding to control RNA sequences shows no enrichment for RNA processing factors.** Gene ontology (GO) enrichment analysis of proteins showing higher affinity for (A) UpA-low and (B) artificial UpA-low (UpA-low_a) sequences compared to UpA-high RNA in uninfected and Zika virus (ZIKV)-infected A549 cell lysates. Analysis includes biological process, molecular function, and cellular compartment categories. Terms are plotted according to their signal strength (x-axis), which combines the enrichment effect size (observed/expected ratio) and statistical significance (-log10 FDR). Circle size indicates the number of proteins in each term. GO terms with more than 80% semantic similarity are clustered and shown in the same colour to reduce redundancy.

**Supplementary Figure 5. Clustered STRING analysis of the proteins binding with high affinity to UpA-high RNA sequences independent of infection status.** Protein-protein interaction network of the 49 proteins showing enhanced binding to UpA-high RNA compared to UpA-low and UpA-low_a sequences in both uninfected and infected conditions. Markov Clustering (MCL) was performed to identify functional groups within the STRING network using default settings.

**Supplementary Figure 6. Analysis of differential protein binding between WNV 3' UTR and NS2A sequences.** Volcano plot comparing protein binding profiles between West Nile virus 3' UTR and NS2A coding sequence. The x-axis shows log10 fold change in protein abundance (3' UTR/NS2A), and the y-axis shows the -log10 of the *p*-value. Statistical significance was determined using a two-tailed Student's t-test. Proteins with significant changes in binding (*p* < 0.05) are shown above the dotted line. Data represent results from three independent RNA affinity purification experiments.

**Supplementary Figure 7. Clustered STRING analysis of the proteins binding with high affinity to West Nile virus 3’ UTR RNA sequences.** Protein-protein interaction network of the significant proteins showing enhanced binding to West Nile virus 3’ UTR RNA compared to NS2A RNA sequences. Markov Clustering (MCL) was performed to identify functional groups within the STRING network using default settings.

**Supplementary Figure 8. Gene ontology analysis of proteins differentially binding to WNV 3' UTR and NS2A sequences.** Gene ontology (GO) enrichment analysis of proteins showing higher affinity for West Nile virus (WNV) (A) 3' UTR compared to NS2A coding sequence, and (B) NS2A coding sequence compared to 3' UTR in A549 cell lysates. Analysis includes biological process, molecular function, and cellular compartment categories. Terms are plotted according to their signal strength (x-axis), which combines the enrichment effect size (observed/expected ratio) and statistical significance (-log10 FDR). Circle size indicates the number of proteins in each term. GO terms with more than 80% semantic similarity are clustered and shown in the same colour to reduce redundancy.

**Supplementary Figure 9. Cytotoxicity assessment of splicing inhibitors in Vero cells.** Cell viability of Vero cells treated with different concentrations of (A) SRPIN340, (B) ISOGK, or (C) splitomicin. Cells were incubated with the indicated concentrations of inhibitors for 72 hours, after which cell viability was assessed using an MTT assay. Data are presented as percentage viability relative to DMSO-treated control cells (mean ± SEM from three independent experiments). (D) Validation of splicing inhibition efficacy by qPCR analysis of GAPDH transcripts. Equal RNA amounts from treated and untreated cells were analysed using exon-exon junction-spanning primers. Relative spliced transcript quantities were calculated based on GAPDH Cq values, confirming the inhibitory effect of each compound on cellular splicing. Statistical significance was determined using a non-parametric Friedman test with Dunn's multiple (**p* < 0.05, ** *p* < 0.01).

**Supplementary figure 10. Northern blot analyses of sfRNA.** Northern blot analysis of subgenomic flavivirus (sf)RNA production of WNV following treatment with the various splicing pathway inhibitors. Numbers represent the average signal intensities for sfRNA1 and sfRNA2 relative to DMSO treated samples. Two biological replicates are loaded on the same gel, separated by the dashed line.
